# Quantification of Head Tremors in Medical Conditions: A Comparison of Analyses Using a 2D Video Camera and a 3D Wireless Inertial Motion Unit

**DOI:** 10.3390/s22062385

**Published:** 2022-03-19

**Authors:** David Amarantini, Isabelle Rieu, Giovanni Castelnovo, Frédérique Fluchère, Chloé Laurencin, Bertrand Degos, Aurélia Poujois, Alexandre Kreisler, Sophie Sangla, Mélissa Tir, Isabelle Benatru, Geneviève Blanchet-Fourcade, Dominique Guehl, Dominique Gayraud, Laurent Tatu, Christine Tranchant, Franck Durif, Marion Simonetta-Moreau

**Affiliations:** 1 Toulouse NeuroImaging Center (ToNIC), Inserm, UPS, Université de Toulouse, 31024 Toulouse, France; simonetta.m@chu-toulouse.fr; 2 Department of Neurology, CNRS, CHU Clermont-Ferrand, Institut Pascal, Université Clermont-Auvergne, 63001 Clermont-Ferrand, France; irieu@chu-clermontferrand.fr (I.R.); franck.durif@uca.fr (F.D.); 3 Service de Neurologie, Centre Hospitalier Universitaire Caremeau, 30029 Nîmes, France; giovanni.castelnovo@chu-nimes.fr; 4 Department of Neurology and Movement Disorders, Timone Hospital, Aix-Marseille Université, 13005 Marseille, France; frederique.fluchere@ap-hm.fr; 5 Université de Lyon, Université Claude Bernard Lyon 1, Lyon Neuroscience Research Center, INSERM, U 1028, CNRS, UMR 5292, Neuroplasticity and Neuropathology of Olfactory Perception team, 69000 Lyon, France; chloe.laurencin@chu-lyon.fr; 6 Service de Neurologie C, Pierre Wertheimer Neurological Hospital, Hospices Civils de Lyon (HCL), 69000 Lyon, France; 7 Service de Neurologie, APHP, Hôpital Avicenne, Université Sorbonne Paris Nord, 93000 Bobigny, France; bertrand.degos@aphp.fr; 8 Dynamics and Pathophysiology of Neuronal Networks Team, Center for Interdisciplinary Research in Biology, Collège de France, CNRS UMR7241/Inserm U1050, Université Paris Sciences & Lettres (PSL), 75005 Paris, France; 9 Neurology Department, Rothschild Foundation Hospital, 75019 Paris, France; apoujois@for.paris; 10 Service de Neurologie A, Movement Disorders Unit, CHU Lille, 59037 Lille, France; alexandre.kreisler@chru-lille.fr; 11 Unité Parkinson, Hôpital Fondation Rothschild, 75019 Paris, France; ssangla@for.paris; 12 Department of Neurology and the Department of Neurosurgery, Expert Centre for Parkinson’s Disease, Amiens University Hospital, 80054 Amiens, France; tir.melissa@chu-amiens.fr; 13 EA 4559 Laboratoire de Neurosciences Fonctionnelles et Pathologie (LNFP), University of Picardy Jules Verne (UPJV), 80054 Amiens, France; 14 Department of Neurology, University Hospital of Poitiers, 86000 Poitiers, France; isabelle.benatru@chu-poitiers.fr; 15 INSERM, CHU de Poitiers, University of Poitiers, Centre d’Investigation Clinique CIC1402, 86000 Poitiers, France; 16 Service de Neurologie, Narbonne Hospital Centre, 11100 Narbonne, France; genevieve.blanchet-fourcade@ch-narbonne.fr; 17 Centre Hospitalier Universitaire de Bordeaux, Institut des Maladies Neurodégénératives, CNRS, University of Bordeaux, 33000 Bordeaux, France; dominique.guehl@u-bordeaux.fr; 18 Service de Neurologie, Centre Hospitalier Intercommunal Aix-Pertuis, Site d’Aix-en-Provence, Avenue des Tamaris, 13616 Aix-en-Provence, France; dgayraud@ch-aix.fr; 19 Department of Neuromuscular Diseases and Department of Anatomy, CHRU Besançon, University of Franche-Comté, 25030 Besançon, France; laurent.tatu@univ-fcomte.fr; 20 Department of Neurology, CHU Hautepierre, 67000 Strasbourg, France; christine.tranchant@chru-strasbourg.fr; 21 Department of Neurology, University Hospital of Toulouse, 31300 Toulouse, France; 22 Clinical Investigation Center (CIC 1436), Toulouse University Hospital, INSERM, 31059 Toulouse, France

**Keywords:** head tremor amplitude, head tremor frequency, 2D video motion analysis, miniature wireless inertial magnetic motion unit, Fourier transform, wavelet transform

## Abstract

This study compares two methods to quantify the amplitude and frequency of head movements in patients with head tremor: one based on video-based motion analysis, and the other using a miniature wireless inertial magnetic motion unit (IMMU). Concomitant with the clinical assessment of head tremor severity, head linear displacements in the frontal plane and head angular displacements in three dimensions were obtained simultaneously in forty-nine patients using one video camera and an IMMU in three experimental conditions while sitting (at rest, counting backward, and with arms extended). Head tremor amplitude was quantified along/around each axis, and head tremor frequency was analyzed in the frequency and time-frequency domains. Correlation analysis investigated the association between the clinical severity of head tremor and head linear and angular displacements. Our results showed better sensitivity of the IMMU compared to a 2D video camera to detect changes of tremor amplitude according to examination conditions, and better agreement with clinical measures. The frequency of head tremor calculated from video data in the frequency domain was higher than that obtained using time-frequency analysis and those calculated from the IMMU data. This study provides strong experimental evidence in favor of using an IMMU to quantify the amplitude and time-frequency oscillatory features of head tremor, especially in medical conditions.

## 1. Introduction

Head tremor can generate involuntary movements of the head in 3D space, either in isolation (focal), in association with limbs tremor (essential tremor), or combined with dystonia (dystonic tremor), according to the new classification [1]. The severity of head tremor can be estimated by clinical rating scales of sub-items [2,3,4,5]. The clinimetric properties of these scores—especially in terms of reliability, validity, and sensitivity to change—seem acceptable, although not perfect (for a review, see [6]). With direct application in clinical environments where repeatable tremor measurement devices could be useful to set up anti-tremoric therapeutic trials and corroborate the results of clinical ratings, complementary computerized tremor quantification and monitoring methods have been developed using either video-based motion analysis systems or more wearable devices, such as wireless inertial units [7]. However, most of these tremor quantification tools concern the upper limbs, [8,9,10,11,12] and, although some studies have focused on head tremor measurements using inertial sensors [13,14,15,16,17,18], the advantages of such wearable sensors for the assessment of head tremor in medical conditions are yet to be determined.

The aim of this study was thus to compare, in a group of patients with head tremor, two different head movement quantification methods: one based on 2D video-based motion analysis, and the other one using a miniature wireless inertial magnetic motion unit (IMMU). To that end, the detection of changes in kinematic head movements (amplitude, frequency) using both systems was analyzed in forty-nine patients in order to select the most sensitive and accurate method for use in further therapeutic head tremor trials. Correlations between clinical scores and signal recordings were also investigated. The purpose was to know if the data collected by each method were complementary, redundant, or presented more advantages than the other. Further, we investigated the advantage of a time-frequency analysis compared to the classical Fourier transform (FFT) for tremor frequency measurement, given the non-stationarity of the signals characterized by “a time-varying behavior of the tremor amplitudes and frequencies” [19].

## 2. Materials and Methods

### 2.1. Participants

The baseline data recorded from the first forty-nine patients with a head tremor enrolled in a clinical trial of Btx-HT protocol (see Institutional Review Board Statement) were analyzed in this methodological ancillary study (age: 64.5 ± 0.22 years; males: 8 (16.3%); time since diagnosis: 15.9 ± 0.33 years). To be included, the patients had to be 18–80 years old and should present a head tremor associated with limbs tremor (essential tremor) or without limbs tremor (focal head tremor). Patients with a head tremor combined with a dystonic component, according to the Tsui scale (>1), or with cerebellar tremor were excluded from the study. The head tremor must have been troublesome (a score of ≥2 for head tremor severity item on the Fahn-Tolosa-Marin tremor rating scale [4] performed at rest when sitting at baseline).

### 2.2. Clinical Assessment of Head Tremor Severity

For each patient, head tremor severity (TOTHEADTREM) was scored (0–8), just before head movement quantification using the “head” sub-items of the Fahn-Tolosa-Marin tremor rating scale (TRS), as the sum of the scores obtained at rest when lying down (0–4) and with posture holding when sitting (0–4).

### 2.3. Materials

**Figure 1:**
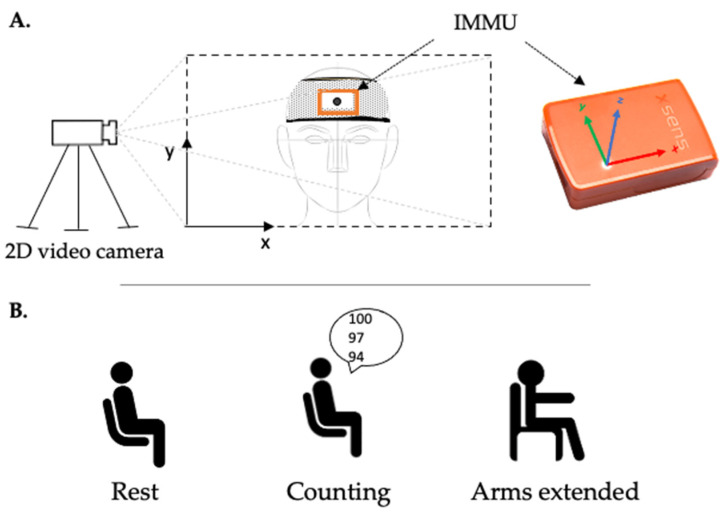
Schematic representation of the experimental setup. (**A**) The IMMU was mounted on the forehead using a disposable headband above the eyebrows to measure the angular displacements of the head. A video camera was placed on a tripod in front of the patient in order to focus on the participant’s face. A black marker on a white background was positioned over the IMMU to track the two-dimensional linear displacements of head motion by the camera. (**B**) Video and IMMU data were simultaneously collected in three successive conditions: at rest in a sitting position for 60 s, for 30 s of backward counting aloud, and with arms outstretched against gravity (arms extended) for 30 s.

For each patient in each center, head movements were simultaneously recorded with a wireless inertial magnetic measurement unit (IMMU) (MTw Awinda sensor, Xsens Technologies BV, Enschede, Netherlands; see [20] and https://www.xsens.com/products/mtw-awinda (accessed on 10 March 2022) for detailed technical specifications) and with one video camera (Figure 1).

The MTw Awinda IMMU is a miniature (dimensions: 34.5 × 57.8 × 14.5 mm; weight: 27 g) motion tracker (MT) designed for highly accurate 3D kinematic applications that include a tri-axis accelerometer, a gyroscope, and a magnetometer in one package [20]. It was mounted on the forehead using a disposable headband above the eyebrows so that the angular displacements around the x, y, and z axes could be taken to represent forward–backward bending (roll), left–right rotation (pitch), and lateral bending (yaw), respectively.

For the video recordings, each experimenter was asked to place a camera with a minimal video resolution of 1280 × 720 pixels (i.e., standard HD) on a tripod in front of the patient in a well-lit room, and to focus the camera on the participant’s face. A black marker (diameter: 10 mm) on a white background was positioned over the IMMU in the middle of the forehead to track the two-dimensional linear displacements of head motion by the camera. Referring the frontal plane, the x-axis represented the medio-lateral direction, while the y-axis represented the vertical direction.

### 2.4. Procedure

To obtain a better comparison of the tremor measurements, video and IMMU data were simultaneously collected in three successive and different conditions (Figure 1):

1. In a sitting position for 60 s (sitting condition);
2. Counting backward, aloud, for 30 s while sitting (counting condition);
3. With arms outstretched, against gravity, for 30 s while in a sitting position (arms extended condition).

### 2.5. Data Processing

For data processing, all the computations were done using MATLAB (MathWorks, Natick, MA, USA). All the filters used were fourth-order, zero-lag Butterworth-type.

#### 2.5.1. Data Preprocessing

For each subject and each experimental condition, the two-dimensional linear displacements of the marker in the frontal plane (x and y along respectively horizontal and vertical axes) were obtained at 25 frames per second from video data using the FaceTracking software (Viewpoint, Lyon, France; version 1.4). Angular position (roll, pitch, and yaw around the x-axis, y-axis, and z-axis, respectively) was obtained at 100 Hz from MTw data using MT Manager software (MT Software Suite, Xsens Technologies BV, Enschede, Netherlands). The times series of raw linear displacements from the video camera or angular displacements from the IMMU were then high-pass-filtered at 1 Hz to selectively remove artifacts while preserving the frequency components of the pathological tremor [21].

#### 2.5.2. Data Transformation

**Figure 2:**
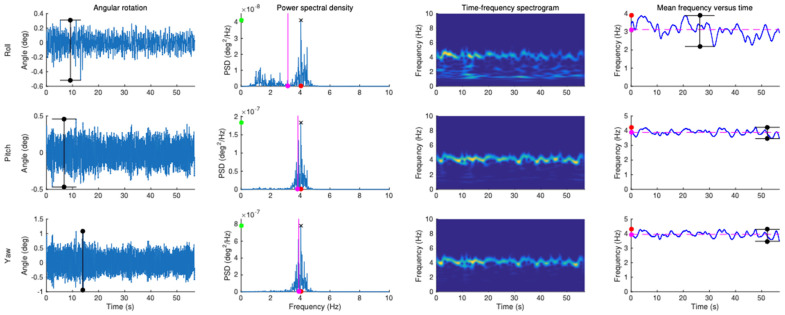
Typical recordings and illustrations of data processing and examples for the angular analysis of head tremor obtained from the IMMU data for forward–backward bending (roll, around x axis), left–right rotation (pitch, around y axis), and lateral bending (yaw, around z axis) in the sitting condition. On each angular rotation plot, the line between black dots represents the angular range of head tremor. On each power spectral density plot, the pink dot corresponds to the mean head tremor frequency, while the red and green dots represent respectively the value and the peak power of the dominant frequency of head tremor identified by a cross. On each plot of mean frequency vs. time, the line between black dots represents the range of head tremor frequency, while the pink and red dots represent respectively the average mean frequency and maximum head tremor frequency.

After preprocessing, data transformation involved the following analyses (see Figure 2 for an illustration of IMMU data and Table 1 for the list of the dependent variables used in the study after transformation):

- Amplitude of head tremor (HTA): For each linear component of head tremor obtained from the video camera (i.e., x, y) and each angular component of head motion obtained from the IMMU (i.e., roll, pitch, and yaw), the amplitude of head tremor was quantified using time domain analysis by the peak-to-peak value of the corresponding filtered displacement signal.
- Head tremor frequency: For each linear component of head tremor obtained from the video camera (i.e., x, y) and each angular component of head motion obtained from the IMMU (i.e., roll, pitch, and yaw), the frequency of head tremor was quantified using both frequency domain and time-frequency domain analyses. In comparison to frequency domain analysis, time-frequency domain analysis allows simultaneously extracting the temporal and spectral information contained in the signals analyzed. It makes it possible to determine which frequencies are present in the signal at a particular time, and thus takes into account non-stationary signals whose frequency behavior changes with time.
  1. Frequency domain analysis: the head tremor frequency calculated in the frequency domain (HTF-F) was defined as the mean frequency of the power spectrum of the filtered displacement signal obtained by using a fast Fourier transform (FFT).
  2. Time-frequency domain analysis: the head tremor frequency calculated in the time-frequency domain (HTF-TF) was defined as the average over time of the mean frequency obtained at each time instant from the time-frequency power spectrum of the filtered displacement signal. The time-frequency transform was performed using Morlet wavelet with the WavCrossSpec software [22] adapted from the MATLAB package developed by [23]. In WavCrossSpec, the scale resolution of the wavelet (parameter ‘nvoice’), the number of scales used in the wavelet analysis (parameter ‘J1′), and the Morlet mother wavelet parameter (parameter ‘wavenumber’) were set to 50, 3.92, and 16, respectively, to provide a satisfactory compromise between time and frequency resolution for the analysis of the spectral content of head tremor.

### 2.6. Statistics

All the values were normally distributed (Shapiro-Wilk test; *p* > 0.05) and met the assumption of homogeneity of variances (Levene’s test, all *p* > 0.05).

Two-factor condition × axis analysis of variance (ANOVA) with repeated measures on all the factors was conducted on head tremor amplitude separately for linear and angular displacements, with the unit being different in both cases and making the comparison between the methods unfeasible (the linear displacements x and y are stated in meters, while the angular displacements, roll, pitch, and yaw, are in degrees, Table 1). The condition factor had three levels (sitting, counting, and arms extended), and the axis factor had two levels (x-axis and y-axis) for linear displacements and three levels (x-axis, y-axis, and z-axis) for angular displacements.

Three-factor condition (sitting vs. counting vs. arms extended) × method (video vs. IMMU) × analysis (frequency domain vs. time-frequency domain) ANOVA with repeated measures on all factors was conducted on head tremor frequency calculated for the axis along/around which tremor amplitude was maximum for each method. The rationale for this design was to select the most appropriate design to test the effects of the method and analysis on the frequency estimation of head tremor, while avoiding a four-factor analysis, which makes the interpretation of higher-order interactions complex.

Table 1 lists the dependent variables used in the study after data transformation and provides accompanying definitions.

Additionally, Spearman rank analyses were used to investigate the link between the severity of head tremor (TOTHEADTREM) and the resulting linear amplitude of head tremor obtained from video data, as well as the angular distance (i.e., the sum of the angles) of head tremor obtained from the IMMU data. The values of the resulting linear amplitude and angular distance of head tremor were log-transformed to account for the non-linear nature of the relationship between the transducer measures of tremor and the rating scores [14,24,25].

**Figure 3:**
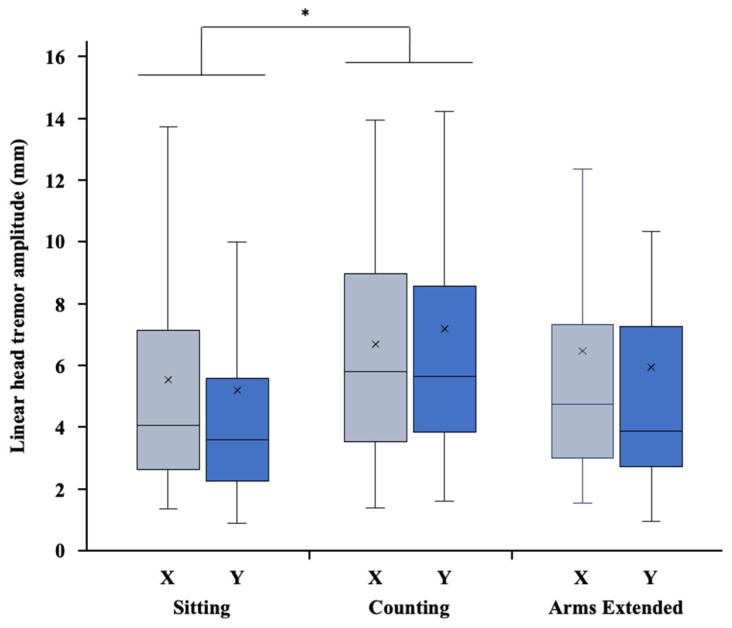
Box plots of the linear head tremor amplitude obtained from video data along the x (X, horizontal) and y (Y, vertical) axes in the sitting, arms extended, and counting conditions. In each box, the center line and the cross respectively represent the median and the mean value, the top and bottom of the box correspond to the 25th and 75th percentiles, and the whiskers represent the 10th and 90th percentiles. Note: * indicates a significant difference between conditions (*p* < 0.05).

**Figure 4:**
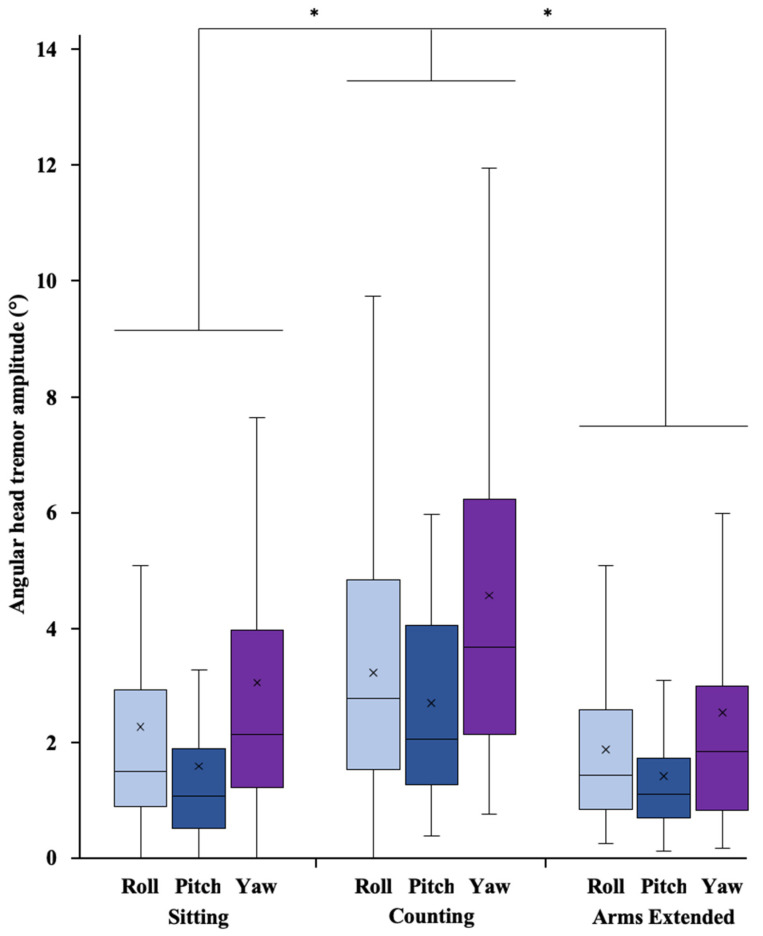
Box plots of the angular head tremor amplitude obtained from the IMMU data around the x (forward–backward bending, roll), y (left–right rotation, pitch), and z (lateral bending, yaw) axes in the sitting, counting and arms extended conditions. In each box, the center line and the cross represent the median and the mean value, respectively; the top and bottom of the box correspond to the 25th and 75th percentiles; and the whiskers represent the 10th and 90th percentiles. Note: * indicates a significant difference between conditions; all pairwise differences among the axes were significant (*p* < 0.05).

**Figure 5:**
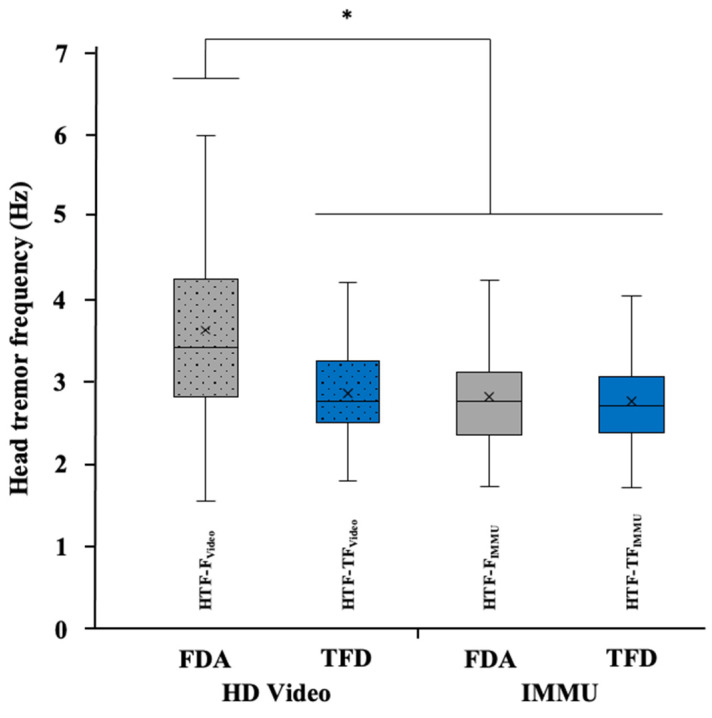
Box plots of the head tremor frequency obtained from the video and IMMU data using frequency domain analysis (FDA) and time-frequency domain analysis (TFD). In each box, the center line and the cross represent the median and the mean value, respectively; the top and bottom of the box correspond to the 25th and 75th percentiles; and the whiskers represent the 10th and 90th percentiles. Note: * indicates a significant difference (*p* < 0.05).

Data are reported as mean ± SD within the text and as mean ± SE in Table 2 and in Figure 3, Figure 4 and Figure 5. Significance was set at *p* < 0.05 for all the analyses.

## 3. Results

### 3.1. Clinical Severity of Head Tremor (TOTHEADTREM)

The mean clinical severity of total head tremor of the forty-nine patients was 3.04 ± 0.02 (0.76 ± 0.01 at rest when lying down; 2.29 ± 0.01 when sitting).

### 3.2. Amplitude of Head Tremor (HTA)

The two-factor ANOVA performed on the linear amplitude of head tremor obtained from the video data (Figure 3) revealed a significant effect of condition (F_2,96_ = 4.070, *p* = 0.020, η^2^_p_ = 0.078), but did not reveal an axis effect (F_1,48_ = 0.090, *p* = 0.765, η^2^_p_ = 0.002) or a significant interaction between condition and axis (F_2,96_ = 1.507, *p* = 0.227, η^2^_p_ = 0.030).

Post hoc comparisons using the Bonferroni correction showed that linear HTA was higher in the counting condition than in the sitting condition (mean difference: 1.57 mm, t_96_ = 2.85, *p* = 0.016), but there was no significant difference between the counting and the arms extended conditions (t_96_ = 1.31, *p* = 0.577) or between the sitting and the arms extended conditions (t_96_ = 1.54, *p* = 0.383).

The two-factor ANOVA performed on the angular amplitude of head tremor obtained from the IMMU data (Figure 4) revealed a significant effect of condition (F_2,96_ = 20.29, *p* < 0.001, η^2^_p_ = 0.299) and a significant effect of axis (F_2,96_ = 20.52, *p* < 0.001, η^2^_p_ = 0.297), but did not reveal a significant interaction between condition and axis (F_4,192_ = 1.58, *p* = 0.181, η^2^_p_ = 0.032).

Post hoc comparisons using the Bonferroni correction showed that angular HTA was higher in the counting condition than in both the sitting (mean difference: 1.259°, t_96_ = 4.72, *p* < 0.001) and the arms extended (mean difference: 1.618°, t_96_ = 6.07, *p* < 0.001) conditions, while no significant difference was found on angular HTA between sitting and arms extended conditions (t_96_ = 1.35, *p* = 0.542). Moreover, post hoc tests revealed that all the pairwise differences between the axes were statistically significant, with mean differences of angular HTA being 0.636° between roll and pitch (t_96_ = 2.77, *p* = 0.020), 0.831° between yaw and roll (t_96_ = 3.62, *p* = 0.001), and 1.467° between yaw and pitch (t_96_ = 6.39, *p* < 0.001). Thus, regardless of the condition, the IMMU analysis distinguished HTA between forward–backward bending (roll), left–right rotation (pitch), and lateral bending (yaw), with the highest angular HTA of 3.38 ± 1.05° obtained for lateral bending around the z-axis (vs. 2.49 ± 0.72° for forward–backward bending around the x-axis and 1.91 ± 0.68° for left–right rotation around the y-axis).

### 3.3. Frequency of Head Tremor (HTF)

Regardless of the analysis, the mean frequency of head tremor calculated for the axis along/around which tremor amplitude was maximum was 3.24 ± 0.92 Hz from video data and 2.78 ± 0.60 Hz from the IMMU data. The three-factor ANOVA performed on the frequency of head tremor revealed a significant effect of condition (F_2,96_ = 15.75, *p* < 0.001, η^2^_p_ = 0.247), a significant effect of method (F_1,48_ = 12.75, *p* < 0.001, η^2^_p_ = 0.210), a significant effect of analysis (F_1,48_ = 54.06, *p* < 0.001, η^2^_p_ = 0.530), and a significant interaction between method and analysis (F_1,48_ = 33.96, *p* < 0.001, η^2^_p_ = 0.414).

In view of the mechanistic link between amplitude and frequency, post hoc comparisons using the Bonferroni correction agreed with the results obtained on both linear and angular HTA: HTF was lower in the counting condition than in both the sitting (mean difference: 0.495 Hz, t_96_ = 5.33, *p* < 0.001) and the arms extended (mean difference: 0.390 Hz, t_96_ = 4.19, *p* < 0.001) conditions, while no significant difference was found on HTF between sitting and arms extended conditions (t_96_ = 1.14, *p* = 0.775). More importantly, regarding the methodological concerns raised in the present study, post hoc tests revealed that the head tremor frequency obtained from the video data was higher than that obtained from the IMMU data, regardless of the analysis (mean difference: 0.451 Hz, t_48_ = 3.57, *p* < 0.001). In addition, regardless of the method, the head tremor frequency calculated using a frequency domain analysis was higher than that obtained using a time-frequency domain analysis (mean difference: 0.411 Hz, t_48_ = 7.35, *p* < 0.001). More precisely, the head tremor frequency calculated using frequency domain analysis from the video data (HTF-F_Video_) was significantly higher than HTF-TF_Video_, HTF-F_IMMU_, and HTF-TF_IMMU_ (all *p* < 0.001, Figure 5), without any significant difference between these three latter variables (all *p* > 0.05, Figure 5).

### 3.4. Correlations between the Severity and the Amplitude of Head Tremor

Spearman rank correlation analysis indicated no significant relationship between TOTHEADTREM and the resulting linear amplitude of head tremor obtained from the video data in each of the sitting, arms extended, and counting conditions (all *p* > 0.05, Table 2).

Conversely, the angular distance of head tremor obtained from the IMMU data was significantly correlated with TOTHEADTREM in both the sitting and arms extended conditions (*p* < 0.001, Table 2).

## 4. Discussion

In this work, we compared the video-based and IMMU-based assessment of head movements in forty-nine patients with isolated head tremor or head tremor associated with tremor in any other part, with two aims. The first aim was to examine the convergence of the quantitative experimental assessment of head tremor using a video camera and an inertial measurement unit in association with the clinical assessment of head tremor severity. The second aim was to evaluate the advantages of using a time-frequency approach to assess head tremor characteristics with the view of proposing the most appropriate metric for use in further therapeutic trials. In line with recent studies supporting the relevance of gyroscopic transducers and their use in tremor analysis [13,14,26], our results clearly confirm the greater capacity of the inertial measurement unit in comparison to a video camera to detect changes of tremor amplitude between conditions. Moreover, they plead for the use of time-frequency analysis to properly characterize head oscillatory movements.

The comparison of the changes in amplitude of head tremor measured from both video and IMMU data showed, irrespective of the axes, the main effect of the conditions of examination on head tremor. For both methods, the amplitude of head tremor was higher as the patients were counting backward when compared to that of resting in the sitting condition. This is not surprising and confirms well-known clinical observations of an exacerbation of head tremor while patients are engaged in a mental task [27]. The amplitude of head tremor calculated from the video data with arms extended was not different from that calculated in the two other conditions of examination (i.e., when counting backward and when at rest in a sitting position), whereas it was different between all conditions when assessed from IMMU data. This difference of ability to detect small amplitude changes according to examination conditions between the two methods could first be explained by the greater difficulty in obtaining perfect standardized measurement conditions among the 16 participating centers with video-based kinematic analysis. Although the experimenters paid particular attention to the instructions of the protocol, we cannot exclude that video camera recordings of head tremor kinematics raised more difficulties regarding the standardization of the experimental procedure between the participating centers than did the IMMU recordings, which were performed more easily in a standardized manner by all the centers. These methodological observations suggest that it is more difficult to obtain standardized experimental recordings with a video camera system than with an IMMU device, which could represent an additional problematic variability factor for repeated measures such as those used in therapeutic trials.

In addition, we were not able to find any significant correlation between the TOTHEADTREM score and the video-based head tremor amplitude measurements. On the contrary, and in agreement with the existing literature [25], the angular distance of head tremor obtained from the IMMU data was significantly correlated with the clinical rating score in both the sitting and arms extended conditions.

Taken together, our results are thus in favor of using the inertial measurement unit rather than video camera to measure the variations of amplitude of head tremor with sensitivity, especially in an ambulatory setting. Thus, our results provide the first experimental evidence of the better sensitivity of the 3D inertial measurement unit in comparison to a 2D video camera to detect small changes in tremor amplitude and quantify head tremor, which could represent a considerable advantage for clinical therapeutic applications. Such an advantage can be related to the fact that, as recalled by Elble and McNames [25], “head tremor is primarily rotation of the head about the neck”, thus making angular measurements obtained from an IMMU ideal when compared to linear ones from a video camera.

Data obtained from the different methods to measure the frequency of head tremor further provide interesting results with regards to the second aim of the study. Indeed, a key finding was that the frequency of head tremor calculated from the video data using FFT was significantly higher than that obtained from time-frequency domain analysis and those calculated using the frequency domain and the time-frequency domain from the IMMU data. Whatever the method used in the present study, the mean head tremor frequency obtained for the axis along/around which tremor amplitude was maximum was lower than the ~4.5 Hz previously reported [18]. However, the range of head tremor frequency reported by Wissel et al. [18] was much larger than what we observed, and the apparent discrepancy between these findings can probably be related to the difference in the number of patients with a pure head tremor (*n* = 14 in [18] vs. *n* = 49 in the present study). The significant differences of frequency of head tremor measures we found between the video camera and the IMMU call into question the confidence that can be given to the frequency domain video-based estimation of head tremor frequency. Our results suggest that using an analysis that fails to take into account the presence of non-stationary events in head motion [19] signals when using a video-based assessment of head tremor may lead to an overestimation of the oscillatory characteristics of the tremor. This finding can be related to the poorer ability of a video camera to characterize the rotational kinematics of the head when compared to an IMMU, and it advocates for the use of time-frequency domain analysis, at least when head tremor analysis is carried out using video data. This result is in line with previous studies that highlighted the importance of time-frequency analysis for upper limb tremor [28], and it also highlights, for the first time, the interest of its future use for head tremor assessment.

Taking into account all these findings, our results on head tremor frequency lead us to conclude that time-frequency analysis seems essential to properly characterize the oscillatory characteristics of head tremor when using video-based assessment. Although head tremor frequency was not different between frequency domain and time-frequency domain analysis, time-frequency analysis can be recommended to account for the non-stationarity of head tremor when using accelerometry or IMMU-based assessment. More generally, it is important to underline that time-frequency analysis also offers the opportunity to examine the temporal dynamics of tremor frequency, which could be of interest to assess the effect of an anti-tremoric treatment, irrespective of the method used.

## 5. Conclusions

In conclusion, this study suggests the superiority of the 3D miniature wireless inertial magnetic motion unit (IMMU) in comparison with a 2D video camera system to quantify head tremor amplitude and oscillatory characteristics, with a lower computational and analysis time-cost. Even if IMMU can present sources of errors, such as bias, calibration, and scale factors, measurements of head tremor using a miniature IMMU device appears more sensitive and easier to perform in clinical practice in comparison with a 2D video system, especially in multicenter clinical protocols to detect therapeutic-induced changes. In addition, our results confirm the great interest of measuring head oscillations using time-frequency domain analysis instead of a classical Fourier transform (FFT) frequency domain analysis.

## Figures and Tables

**Table 1:** List of the dependent variables used in the study after data transformation.

Method	Video		IMMU		
Movement type	Translation		Rotation		
Axis	x	y	x	y	z
Measured component of head motion	X (m)	Y (m)	Roll (°)	Pitch (°)	Yaw (°)
Dependent variables for head tremor					
Peak-to-peak head tremor amplitude (HTA)	HTA_X_ (m)	HTA_Y_ (m)	HTA_Roll_ (°)	HTA_Pitch_ (°)	HTA_Yaw_ (°)
Head tremor frequency (HTF, in Hz) *					
Frequency domain	HTF-F_Video_		–	–	HTF-F_IMMU_
Time-frequency domain	HTF-TF_Video_		–	–	HTF-TF_IMMU_

* The analysis on head tremor frequency (HTF) was conducted for the axis along/around which tremor amplitude was maximum for each method.

**Table 2:** Summary of the Spearman rank correlation analysis between head tremor severity (TOTHEADTREM) and log-transformed values of linear and angular amplitude of head tremor.

Model	Condition	R	R^2^	Estimate	SE	t	*p*
Resultant linear amplitude-TOTHEADTREM	Sitting	0.277	0.075	0.072	0.037	1.95	0.058
	Arms extended	0.293	0.048	0.057	0.037	1.54	0.130
	Counting	0.280	0.014	0.026	0.031	0.807	0.423
Angular distance-TOTHEADTREM	Sitting	0.468	0.219	0.192	0.053	3.626	<0.001 *
	Arms extended	0.521	0.272	0.148	0.035	4.19	<0.001 *
	Counting	0.207	0.043	0.057	0.039	1.45	0.154

* indicates a significant relationship between variables (*p* < 0.05).

## Data Availability

Not applicable.
